# Between neurons and networks: investigating mesoscale brain connectivity in neurological and psychiatric disorders

**DOI:** 10.3389/fnins.2024.1340345

**Published:** 2024-02-20

**Authors:** Ana Clara Caznok Silveira, Andre Saraiva Leão Marcelo Antunes, Maria Carolina Pedro Athié, Bárbara Filomena da Silva, João Victor Ribeiro dos Santos, Camila Canateli, Marina Alves Fontoura, Allan Pinto, Luciana Ramalho Pimentel-Silva, Simoni Helena Avansini, Murilo de Carvalho

**Affiliations:** ^1^National Laboratory of Biosciences, Brazilian Center for Research in Energy and Materials, Campinas, Brazil; ^2^School of Electrical and Computer Engineering, University of Campinas, Campinas, Brazil; ^3^Brazilian Synchrotron Light Laboratory, Brazilian Center for Research in Energy and Materials, Campinas, Brazil; ^4^Neuroimaging Laboratory, Neurology Department, University of Campinas, School of Medical Sciences, Campinas, Brazil

**Keywords:** connectivity, mesoscale, NeuroImage, schizophrenia, epilepsy, computer vision, segmentation, deep learning

## Abstract

The study of brain connectivity has been a cornerstone in understanding the complexities of neurological and psychiatric disorders. It has provided invaluable insights into the functional architecture of the brain and how it is perturbed in disorders. However, a persistent challenge has been achieving the proper spatial resolution, and developing computational algorithms to address biological questions at the multi-cellular level, a scale often referred to as the mesoscale. Historically, neuroimaging studies of brain connectivity have predominantly focused on the macroscale, providing insights into inter-regional brain connections but often falling short of resolving the intricacies of neural circuitry at the cellular or mesoscale level. This limitation has hindered our ability to fully comprehend the underlying mechanisms of neurological and psychiatric disorders and to develop targeted interventions. In light of this issue, our review manuscript seeks to bridge this critical gap by delving into the domain of mesoscale neuroimaging. We aim to provide a comprehensive overview of conditions affected by aberrant neural connections, image acquisition techniques, feature extraction, and data analysis methods that are specifically tailored to the mesoscale. We further delineate the potential of brain connectivity research to elucidate complex biological questions, with a particular focus on schizophrenia and epilepsy. This review encompasses topics such as dendritic spine quantification, single neuron morphology, and brain region connectivity. We aim to showcase the applicability and significance of mesoscale neuroimaging techniques in the field of neuroscience, highlighting their potential for gaining insights into the complexities of neurological and psychiatric disorders.

## 1 Introduction

The human brain is a remarkably intricate network composed of billions of neurons, encompassing diverse cell types interconnected through trillions of synapses (Luo et al., 2008). Different brain regions exhibit distinct microstructural architectures, functional specializations, interconnectivity, and often an orderly topographic arrangement. The major task in connectivity-related research is capturing the hierarchical multiscale organization of the brain by mapping network relationships across various spatial dimensions (Sporns, 2013). It extends beyond structural considerations and encompasses functionality, denoted by the degree of correlation and covariance among brain signals, influenced by both experimental parameters and temporal context (Cabral et al., 2017).

The organization of brain connections plays a pivotal role in shaping interactions between different brain areas, giving rise to a multitude of functional networks. Structural data provide the anatomical framework, while functional data reveal how different brain regions work together and respond to various stimuli or tasks. The multimodal correlation of imaging techniques, integrating both structural and functional neuroimaging methods, allows the harnessing of their best features, offering a broader approach and better understanding of brain connectivity (Howard et al., 2023). This multidimensional approach is essential for advancing our knowledge of complex neurological and cognitive processes (Hirsch et al., 2015).

Multiple, albeit subtle, non-physiological shifts in brain organization likely lead to network disorders which encompass a wide range of neurological and psychiatric conditions arising from aberrant neural connections. These include autism spectrum, schizophrenia, attention-deficit/hyperactivity, epilepsy, depression, and anxiety disorders (Kaiser, 2013; Contreras-Rodríguez et al., 2015; Holmes et al., 2023).

Most of the data used to reconstruct brain networks comes from bidimensional (2D) images. However, the correlation between a single cell interacting with the whole neuronal tissue in a tridimensional (3D) manner remains an open problem. This 3D spatial-scale context holds the key to bridging morphological mechanisms and functional outcomes to better understand the complexities of brain connectivity-related disorders. The complex 3D circuits that define brain connectivity comprise a variety of organizational structures and microarchitectures that can be arduous to discern (Sporns et al., 2005), presenting a significant challenge in the field of neuroscience and computational analysis. Additionally, to preserve the volumetric information of the network it can be necessary to work with samples as thick as possible coupling to 3D-imaging techniques, as extensively applied in image-based neuroresearch and diagnosis (Kim et al., 2021).

**FIGURE 1:**
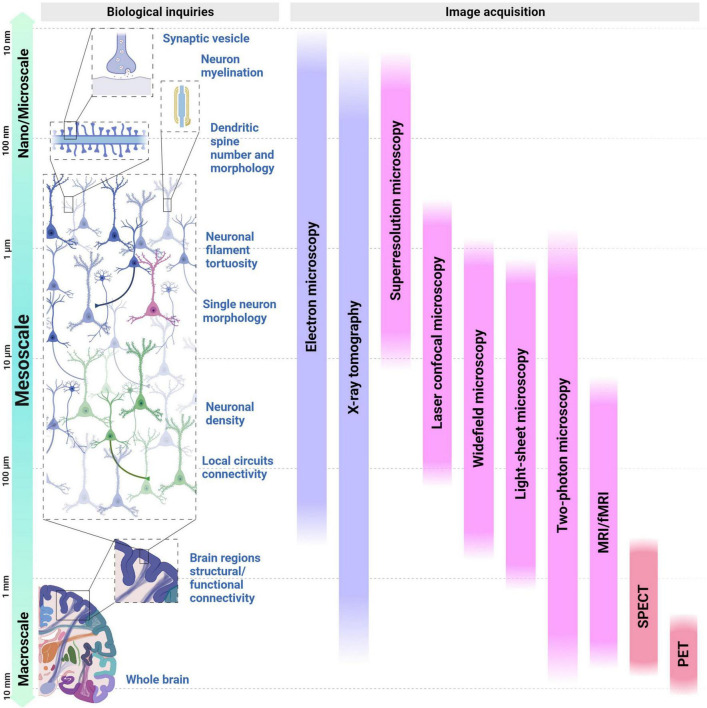
Overview of experimental bioimage tools currently available for studying neural connectivity across a range of spatial scales and biological questions. At the left, different human brain organization structures are presented under the perspective of spatial scales: from the study of the dendritic spine **(top)** to the whole brain **(bottom)**, with a focus on the structures that can be studied using mesoscale imaging. From the **top** to the **bottom**, spatial scales range from 10 nanometers to 10 millimeters. The second section, denoted Biological Inquiries, displays the cellular elements that contribute and shape neural connectivity across the different scales, followed by a repertoire of image acquisition techniques displayed as vertical bars in the last section. Purple bars represent techniques suited to structural imaging while the pink bars represent the ones suited for functional purposes; lastly, the red bars represent techniques that incorporate both. MRI, magnetic resonance imaging; fMRI, functional magnetic resonance Imaging; SPECT, single photon emission computed tomography; PET, positron emission tomography.

The brain connectome *sensu* (Sporns et al., 2005) takes on different definitions at various scales, presenting a defying task in translating morphological and functional measurements to the symptoms of brain disorders affected by connectivity. Understanding integrated brain function demands a multitude of measurements across various scales. Neurophysiological and neuroimaging methods, along with the use of whole-brain models to provide fresh insights into its underlying mechanisms (Hallett et al., 2020). Thus, brain connectivity conventionally encompasses three scales: nano/microscale, mesoscale, and macroscale (Bohland et al., 2009), each one with its optimized imaging method (Figure 1).

At the nano/microscale, lies the ultrastructural information, that can reveal synaptic morphology, their components and connections in individual cells, often employing Electron Microscopy (EM), demanding sample chemical preservation and physical sectioning. The opposite extreme encompasses the macroscale, which examines the anatomical and connective patterns between distinct brain regions, such as long-range connections, often inferred from fiber tracts, and frequently revealed by techniques also capable of retrieving functional aspects, such as Magnetic Resonance Imaging (MRI), Positron Emission Tomography (PET), Single Photon Emission Computed Tomography (SPECT). These approaches prove particularly valuable for non-invasive studies of living tissues (Bennett et al., 2018).

Between both spatial extremities lies the multi-cellular level (Mitra, 2014), also known as the mesoscale, which plays a pivotal role in the investigation of the intricate network of the brain. Mesoscale spans from the structural and functional properties of single neurons to local neural circuits and their intrinsic connectivity (Mitra, 2014; Haueis, 2021).

Most neuroimaging studies on humans and human samples have primarily used macroscale techniques like PET and functional magnetic resonance imaging (fMRI) for *in vivo* imaging, and microscale techniques such as thin-depth light microscopy for tissue samples. Although substantial insights into brain networks and abnormal connectivity have been acquired using these techniques, they lack the spatial resolution needed to resolve the 3D conformation of local neuronal connections (Tyson and Margrie, 2022). Consequently, further progress in the understanding of brain functions within complex neuronal circuits requires exploration at the mesoscale level (Rah et al., 2015). It depicts connections, networks, and spatial cellular gradients of distinct neuronal populations, improving resolution and the analysis of interactions that form the basis of cognitive and behavioral processes (Haueis, 2021). Intact/live samples can be used, albeit sample preparation is necessary according to the specific imaging technique. Optical microscopies (wide field, laser confocal, light sheet, and two-photons) allow both high spatial and temporal resolution, often used to study live cells.

In this landscape, data acquisition and image processing emerge as a critical domain of local neural circuits, i.e., spatially co-localized neurons of the same kind or with shared organizational traits (Bohland et al., 2009). It also generates a huge amount of data to be processed and may not be as easily quantifiable (Lang et al., 2012; Chen et al., 2019). Currently, artificial intelligence algorithms have proved their ability to help researchers in image processing and analysis: from contrast enhancement/normalization to segmentation and extraction of morphological features necessary for structural correlation of connectivity (Durkee et al., 2021).

As stated above, neuroimaging encompasses a diverse array of techniques for exploring different scales of magnitude and activities within cells and tissues. Consequently, data analyses are predominantly linked to the complexities of the images, posing a challenge for neuroscientists who may not be familiar with the intricacies of the field. In this review, we aim to explore mesoscale brain imaging and processing, arranging the main methodologies traditionally used to investigate brain functioning throughout its network. It begins by exploring the state-of-the-art in neurological and psychiatric disorders research and imaging techniques relevant to the field; it then addresses image processing strategies suited to solving these questions. Recent literature was compiled on various imaging modalities to study neural connections and the respective computational methods to identify misorganization in schizophrenia and epilepsy. It also organizes concepts in network neurological disorders to guide non-expert and advanced readers in the field of neuroimaging and processing. Finally, to accommodate the diverse readership in this multidisciplinary field, a Glossary tailored to the terminology of some key concepts in neurobiology, imaging, and computational processing is included.

## 2 Brain connectivity in disorders of the central nervous system

This section explores a selection of connectivity-related issues and the bioimaging techniques employed to address them. Disorders affected by brain connectivity encompass a wide range of neurological and psychiatric conditions arising from aberrant neural connections. These include autism spectrum disorder, schizophrenia, attention-deficit/hyperactivity disorder, epilepsy, depression, and anxiety disorders (Kaiser, 2013; Contreras-Rodríguez et al., 2015; Holmes et al., 2023). Although substantial insights into the network of the brain and abnormal connectivity in these disorders have been gained using macroscale imaging techniques such as MRI and PET, further progress in our understanding requires the exploration at the mesoscale level for increased resolution. In this section, we examine brain connectivity in two different disorders, representing examples from psychiatric and neurological conditions. Our analysis highlights the crucial role of advanced neuroimaging techniques in uncovering the complexities of these conditions. We particularly focus on the potential of mesoscale neuroimaging to further enhance our understanding of their underlying mechanisms.

### 2.1 Schizophrenia

Schizophrenia is a multifactorial mental condition that impacts over 23 million individuals worldwide. It involves positive symptoms such as delusions and hallucinations, negative symptoms such as reduced motivation and social withdrawal, and cognitive impairment. The pathophysiology of schizophrenia involves molecular and morphological abnormalities within the nervous system, encompassing faulty brain connectivity, altered myelination of brain regions and white matter tracts, as well as abnormal neuronal morphology and defects in neurotransmitter systems (Schultz and Andreasen, 1999; Kahn et al., 2015).

Recent years have witnessed significant advancements in imaging studies, shedding light on the neurobiological basis of schizophrenia. In this section, we delve into the contribution of imaging studies to our understanding of the connectivity basis of the disorder.

#### 2.1.1 Structural and functional brain network abnormalities

Coordinated functioning of multiple brain regions is crucial for normal brain function, encompassing perception, cognition, emotions, and mood responses. A significant amount of evidence points to a dysfunctional local circuitry in schizophrenia in the prefrontal cortex (PFC) and its connections with other brain regions, particularly those associated with the limbic system (Lewis et al., 2005). In the past two decades, numerous studies involving neuroimaging techniques like fMRI have yielded compelling findings indicating abnormal activity within the local prefrontal network and disrupted integration of information processes in the PFC and other brain regions among individuals with schizophrenia (Anticevic et al., 2014, 2015; Hunt et al., 2017). Although the evidence supporting disconnectivity in schizophrenia is robust, understanding its causes is complex, and there is ongoing debate regarding its mechanisms and significance concerning clinical symptoms (Gao W. et al., 2022).

Investigations using fMRI have consistently revealed disrupted connectivity in individuals with schizophrenia, both during resting-state conditions and while engaged in specific cognitive tasks (Garrity et al., 2007; Whitfield-Gabrieli et al., 2009; Sheffield and Barch, 2016; Erdeniz et al., 2017; Godwin et al., 2017). More recently, a meta-analysis and an original article reported consistent changes in local functional connectivity in schizophrenia. It was found that patients showed significantly higher Regional Homogeneity (ReHo) in the bilateral medial superior frontal gyrus, while lower ReHo in the bilateral post-central gyrus, right pre-central gyrus, and right middle occipital gyrus (Cai et al., 2022); and differences in the functional connectivity between the salience network and certain brain regions, including the right inferior and middle temporal gyrus, left caudate, and right pre-central gyrus (Huang H. et al., 2022). These findings suggest that there are consistent aberrant local functional connectivity patterns in schizophrenia.

The assessment of functional connectivity in schizophrenia relies predominantly on fMRI scanning data acquired from adult individuals diagnosed with the disorder. However, due to the dependence of fMRI on hemodynamic fluctuations associated with neural activity, it is unsuitable for capturing rapid transitions in brain functional connectivity configurations with high temporal resolution (Jamadar et al., 2021). Moreover, the spatial resolution of this technique is limited to a millimeter scale. As a result, our comprehension of the cellular mechanisms underlying the aberrant brain functional connectivity observed in schizophrenia remains incomplete.

#### 2.1.2 Neurotransmitter systems

Multiple etiological hypotheses have been proposed to elucidate the abnormal brain connectivity seen in schizophrenia. The dopaminergic hypothesis posits that abnormal dopaminergic neurotransmission contributes to the development and manifestation of schizophrenia (Creese et al., 1976; Toda and Abi-Dargham, 2007). Several lines of evidence support the dopaminergic hypothesis such as alterations in dopamine receptor density and availability in affected individuals revealed by PET and SPECT imaging (Patel et al., 2010). Specifically, an increased number of dopamine D2 receptors has been observed in the limbic striatum. Hyperactivity of D2 receptors in the mesolimbic pathway is thought to contribute to the positive symptoms of schizophrenia (Howes et al., 2009).

The glutamatergic hypothesis was also proposed as an additional perspective on the pathophysiology of schizophrenia (McCutcheon et al., 2020). For instance, decreased glutamate levels have been found in the anterior cingulate cortex and prefrontal cortex, regions implicated in cognitive and emotional processing (Chen et al., 2017). Moreover, PET studies have shown abnormalities in the expression, binding, and availability of glutamate receptors in various brain regions of individuals with schizophrenia (Beck et al., 2021).

It is becoming increasingly evident that the dopaminergic and glutamatergic hypotheses alone fall short of providing a comprehensive explanation for the disorder highlighting the need to consider additional neurochemical systems involved in schizophrenia, such as the GABAergic system (Jahangir et al., 2021). fMRI and PET studies have also provided insights into the altered neural connectivity and network dynamics associated with GABAergic abnormalities in the disorder (Shukla et al., 2019; Marques et al., 2021).

#### 2.1.3 Myelin and white matter tracts

Employing MRI, researchers investigated gray/white-matter contrast in sensory and motor regions of the cortex in schizophrenia revealing reduced myelin in three bilateral sensory and motor regions (Jørgensen et al., 2016). Furthermore, a study employing Diffusion Tensor Imaging (DTI-fMRI) observed significantly lower fractional anisotropy (FA) values in white matter tracts of patients with psychosis compared to healthy controls (Xu et al., 2022). Additionally, the study demonstrated a positive correlation between decreased white matter tract integrity and cognitive performance in patients with psychosis. Electron microscopy of brain tissue from individuals with schizophrenia revealed ultrastructural signs of apoptosis and necrosis in oligodendroglial cells within the cortex and the caudate nucleus with damage to myelin sheath lamellae, and a significant decrease in the nucleus area and volume density of mitochondria (Uranova et al., 2001).

#### 2.1.4 Dendritic pathology

Dendritic spines are the primary sites of excitatory synaptic connections (Papa et al., 1995). As such, alterations to their morphology directly impact the neuronal circuitry within and across multiple brain regions, potentially contributing to the pathogenesis of schizophrenia. Studies on schizophrenia subjects have revealed reductions in dendritic spine density, dendritic arborization and plasticity in several cortical and non-cortical areas (Glantz and Lewis, 2000; Konopaske et al., 2014; MacDonald et al., 2017). By employing confocal microscopy, researchers have investigated the formation, maturation, and pruning of synaptic connections, using *in vitro* models (Sellgren et al., 2019). Studies in human stem cell-derived neural models have revealed increased synapse elimination and significant developmental and connectivity issues, including the abnormal spread of proliferating neural progenitor cells from the ventricular zone to the intermediate and cortical zones (Stachowiak et al., 2017). Interestingly, maturing neurons were found to be abundantly developed in the deeper neural structure (analogous to subcortical regions) but were notably depleted in surface layers (analogous to the cortical region) of schizophrenia neural organoids.

### 2.2 Epilepsy

Epilepsy is recognized as a network disorder with multifactorial causes, representing a multiscale challenge that includes cellular, network, and systems levels. It encompasses widespread areas that stretch well beyond the pinpointed site of a seizure, displaying distinctive patterns that might be specific to each particular syndrome (Stafstrom and Carmant, 2015). To gain a comprehensive understanding of the mechanisms underlying hyperexcitability in epilepsy, it is essential to highlight two primary epilepsy classifications. The first is *focal epilepsy*, which is characterized by seizures originating from a specific focal onset within one hemisphere of the brain, as determined by clinical patterns or electroencephalogram (EEG) localization. Common examples of focal epilepsy encompass conditions like mesial temporal lobe epilepsy associated or not with hippocampal sclerosis and malformations of cortical development. The second classification, *generalized epilepsy*, is defined by seizures occurring simultaneously in both hemispheres (Fisher, 2017). In this topic, we review the literature on abnormal neural networks and harness the potential of imaging techniques to address critical knowledge gaps in epilepsy-related brain connectivity.

#### 2.2.1 Structural and functional brain network abnormalities

*In vivo* mapping of the regional distribution of network abnormalities is a crucial way to define precisely the site of seizure onset. The identification of the site where seizures start and how they propagate is critical to understanding both the pathophysiology of epilepsies and developing therapeutic approaches. Macroscale neuroimaging techniques, such as high-resolution MRI and fMRI, are the entrance step in providing insights into the topological organization of brain networks and connectivity disruptions in epilepsy patients.

Extensive findings have emerged from quantitative structural MRI investigations employing volumetry, voxel-based morphometry, cortical thickness mapping, and structural covariance analysis. In DTI investigations, several parameters can be obtained to characterize white matter microstructure including tractography, tensor-derived metrics, and connectivity matrices (Bartolomei et al., 2005).

In structural MRI, volumetric analysis frequently reveals atrophy in limbic structures, such as the hippocampus, entorhinal cortex, and amygdala, which often correlates with histological evidence of neuronal loss in excised temporal lobe epilepsy (TLE) brain tissue (Bartolomei et al., 2005; Bernhardt et al., 2013). Likewise, post-processing methods in quantitative MRI, such as voxel-based morphometry and cortical thickness analysis, have also revealed that TLE is linked to widespread neocortical irregularities. Covariance analyses of these abnormalities extend beyond mesial temporal structures to comprise prefrontal, frontocentral, cingulate, occipitotemporal, and lateral temporal neocortex (Bernasconi et al., 2004; Bernhardt et al., 2012, 2013). ENIGMA-Epilepsy MRI scans showed gray and white matter changes in different epilepsy types, with more widespread and bilateral extra-hippocampal gray matter differences in left TLE (Whelan et al., 2018; Hatton et al., 2020; Sisodiya et al., 2020). Also, in individuals with TLE, the investigation of preoperative structural connectivity using DTI-fMRI and its association with post-operative seizure control outcomes revealed specific preoperative connectivity patterns that are associated with improved surgical outcomes (Bonilha et al., 2013).

While there has been substantial progress in understanding structural connectivity abnormalities at the macroscale, we face limitations due to our access being restricted to network topology without achieving a finer neuronal resolution and specificity. In this regard, the mesoscale provides a more precise comprehension by pinpointing the particular neural components contributing to local connectivity. Thus, the gold standard for noticing abnormal structural connectivity in a mesoscale is anterograde and retrograde viral neuronal tracing (Lanciego and Wouterlood, 2020). These tracers exhibit high accuracy and sensitivity, especially when mapping long-range connections, thus contributing to a comprehensive and detailed understanding of connectivity across various brain areas (Saleeba et al., 2019). Their invasive nature restricts the use to animal models. Du et al. (2017) employed a rat model induced by pilocarpine and utilized rabies tracing techniques to discern intricate morphological details of projections within the dynamic hippocampal circuit. This study revealed that newly formed dentate granule cells (DGCs) in adults, triggered by seizures, receive excitatory signals from pyramidal cells in the cornu Ammonis (CA3) and repeated excitatory inputs from other DGCs.

In fMRI, Englot et al. (2016) explored local and distant synchronization of resting-state fMRI signals in TLE and focal epilepsy patients. They observed altered connectivity within and between various brain regions, highlighting the impact of epilepsy on network organization. Likewise, analysis of resting state in focal cortical dysplasia (FCD) identified distinct patterns of functional connectivity with the hypo-connected patterns in cases with FCD type IIB, whereas the hyperconnected lesions were predominantly associated with type IIA (Hong et al., 2019).

#### 2.2.2 Abnormal neuron morphology

Alterations in the size and shape of neuronal cell bodies have been detected across diverse brain regions, encompassing the hippocampus, neocortex, and other regions linked to abnormal neural connectivity (Stouffer et al., 2016). The connection between these morphological alterations and epileptogenesis has already been confirmed (Abdijadid et al., 2015). These deviations in neuronal cell body structure can influence the interconnection and communication between neurons, potentially influencing the onset and advancement of epilepsy (Hsieh et al., 2016; Wu et al., 2022). More precisely, these alterations in local and global connectivity can impact the manifestation of seizures, determining whether abnormal connectivity and hyperexcitability result in focal or generalized seizures (Sheybani et al., 2018; Represa, 2019).

In focal epilepsies, malformations of cortical development are associated as the primary substrate in which the presence of morphologically abnormal neurons significantly affects neural connectivity (Mainen and Sejnowski, 1996; Richards and Van Hooser, 2018). The existence of atypical neurons could influence the subsequent stages of development that regulate cortical synaptic connectivity (Subramanian et al., 2020). Avansini et al. (2022) observed an enhanced level of network connectivity (termed effective connectivity) along with increased neuronal excitability in human neural organoids derived from pluripotent stem cells of patients with FCD. The aberrant connectivity seen in FCD appears to be influenced by neuronal morphological abnormalities, particularly the presence of dysmorphic neurons. Using 3D confocal microscopy, the researchers detected enlarged cell bodies and increased dendritic complexity, potentially contributing to a more interconnected neural circuitry and the formation of an epileptogenic network in FCD.

Using high-resolution synchrotron x-ray microtomography and Golgi-Cox staining, Fonseca et al. noticed an altered distribution of neurons and a reduction of cell number in the hippocampus in a *status epilepticus* mouse model. These approaches allowed the assessment of the 3D cytoarchitecture, neuron density, and morphology (Fonseca et al., 2018).

#### 2.2.3 Abnormal neuronal localization

The integration of dendrites and synapses into functional networks is heavily affected by how neocortical neurons are positioned during development (Martineau et al., 2018). Malpositioned neurons in the cortex cytoarchitecture are called heterotopic neurons (Ishii et al., 2015). These neurons alone may play a role but do not seem to be sufficient to trigger seizures (Aghakhani et al., 2005). The aberrant organization of cortical cytoarchitecture potentially leads to aberrant connections within these developing neuronal networks. Additionally, the recruitment of distinct microcircuits from different cortical locations could alter synchronicity, leading to abnormal neural oscillations (Dubeau et al., 1995; Abdijadid et al., 2015).

Neuronal disorganization and clusters of heterotopic neurons are primarily observed in human specimens from cortical migration malformations such as periventricular heterotopia (Ekşioğlu et al., 1996) and FCD type I (Coras et al., 2021) using light microscopy with immunohistochemical and DiI tracing techniques. Additionally, in animal epilepsy models (Mello et al., 1993), there have been observations of heterotopic granule cells in the dentate gyrus, resembling those found in human epilepsy. Heterotopic granule cells establish new connections and potentially impact synaptic reorganization (Babb, 1991).

#### 2.2.4 Dendritic pathology

The presence of dendritic spine pathologies and abnormal dendritic arborization have been suggested to be implicated in epilepsy worsening, increasing neuronal hyperexcitability in the circuits, and contributing to cognitive deficits, synaptic remodeling, and aberrant plasticity (Fiala et al., 2002).

Dendritic spines are mostly observed in excitatory synapses and neurons respond to epileptogenic changes in the circuitry by modifying the structure of their dendritic trees. Alterations in the distribution, quantity, and morphology of dendritic spines have been proposed to have a direct impact on seizures and epileptogenesis (Jiang et al., 1998; Jean et al., 2023). However, it remains unclear whether these changes are the cause or are a consequence of seizure recurrence (Wong and Guo, 2013). Dendritic pathology in epilepsy can be broadly categorized into two main fields, as described below:

*Neuronal dendritic arborization*: Morphological changes of dendrites can affect neuronal excitability. Abnormalities in dendritic length, shape, and branching patterns have been described in epilepsies associated with either hippocampal sclerosis, or tumors, or microdysgenesis (von Campe et al., 1997), and also associated with the presence of varicose swelling of the dendrites of granular dentate neurons of the hippocampus (Blümcke et al., 1999).

*Dendritic spine pathology:* The initial observation of dendritic spine loss occurred in hippocampal pyramidal neurons and dentate granule cells among individuals with TLE (Scheibel et al., 1974), providing a plausible mechanism to elucidate the learning and memory challenges experienced by these patients (Chen et al., 2010). In Lennox-Gastaut syndrome, a childhood epileptic disorder linked to intellectual disability, pyramidal neurons from brain biopsy were observed to possess a reduced number of spines using EM (Renier et al., 1988). In human cerebral cortices derived from FCD patients, a reduction of dendritic spines, and sporadic filopodia-like protrusions emerging from the soma in dysmorphic neurons were noticed using Golgi impregnation and confocal microscopy (Rossini et al., 2023).

Employing Golgi-Cox staining, optionally combined with immunohistochemistry, as well as DiI tracing, and utilizing both confocal microscopy and EM techniques, provides a comprehensive method for assessing the morphology and structure of dendritic arborization, as well as the density and morphology of neuronal dendritic spines in epilepsy.

### 2.3 Exploring connectivity in central nervous system disorders via mesoscale imaging for deeper insights

Functional and structural imaging studies have consistently identified aberrant connectivity as a fundamental feature in the pathogenesis of various brain disorders. These investigations have primarily involved live human subjects and focused on a macroscale level, employing techniques such as MRI/fMRI and PET/SPECT, which deliver the overall spatial context of a large field of views, albeit at lower resolution. As seen in Table 1, which compiles brain connectivity studies in schizophrenia and epilepsy from the literature, there has historically been an over-representation of use of macroscale techniques to try to answer biological questions. While these studies have provided valuable insights into the presence of aberrant connectivity, they have fallen short in uncovering its precise etiological underpinnings in different brain disorders. Mesoscale imaging provides a means to address the potential untapped source of information for novel insights pertaining to brain connectivity, as observed in this context.

**TABLE 1:** Compilation of brain connectivity studies in schizophrenia and epilepsy: synthesis across different scales, data acquisition modalities, and image processing strategies.

Biological question	Scale	Data acquisition	Output	Image processing	References
Structural and functional brain network abnormalities	Macroscale	MRI	Functional connectivity	Seg.: in house MATLAB tools	Anticevic et al. (2015)
Functional connectivity	Macroscale	MRI	Task-based functional connectivity	Seg.: registration to neuroanatomical atlas	Garrity et al. (2007)
Functional connectivity	Macroscale	MRI	Task-based functional connectivity	Prep.,^1^ seg.: registration to neuroanatomical atlas coordinates	Whitfield-Gabrieli et al. (2009)
Functional connectivity	Macroscale	MRI	Functional connectivity	Prep.,^1^ seg.: registration to neuroanatomical atlas coordinates	Erdeniz et al. (2017)
Functional connectivity	Macroscale	MRI	Intra- and inter-network task-based functional connectivity	Prep.,^1^ seg.: cortical parcellation of functional connectivity boundaries maps	Godwin et al. (2017)
Functional connectivity	Macroscale	MRI	Functional connectivity	Prep.,^1^ seg.: voxel-wise meta-analysis–SDM-PSI software	Cai et al. (2022)
Functional connectivity	Macroscale	MRI	Functional connectivity	Prep.,^1^ seg.: independent component analysis–CONN toolbox	Huang H. et al. (2022)
Neurotransmitter systems	Macroscale	PET	18F-DOPA uptake	Prep.,^1^ seg: semi-automatic, probabilistic registration to neuroanatomical atlas	Howes et al. (2009)
Neurotransmitter systems	Macroscale	MRI	Glu and GABA levels	Metabolite quantification. Voxel seg: not detailed	Chen et al. (2017)
Neurotransmitter systems	Macroscale	PET	NMDAR ligand tracer volume distribution	Prep.,^1^ seg.: neuroanatomical atlas registration	Beck et al. (2021)
Neurotransmitter systems	Macroscale	MRI	Glu and GABA levels and functional connectivity	Prep.,^1^ seg.: automatic metabolite quantification; functional connectivity in MRS voxel	Shukla et al. (2019)
Neurotransmitter systems	Macroscale	PET	GABAAR ligand tracer volume distribution	Prep.,^1^ seg.: neuroanatomical atlas registration	Marques et al. (2021)
Myelin and white matter tracts	Macroscale	MRI	GM/WM contrast	Seg.: surface-based mapping–FreeSurfer 5.3.0	Jørgensen et al. (2016)
Myelin and white matter tracts	Micro/Nanoscale	EM	Myelin sheath lamellae damage	Seg.: manual analysis–Kontron Mop–Videoplan image analyzer	Uranova et al. (2001)
Dendritic spine quantification	Micro/Nanoscale	LM	Mean diameter, total length, location and number of dendritic spines	Manual tracing	Glantz and Lewis (2000)
Dendritic spine quantification	Micro/Nanoscale	BM	Spine density and dendrite length	Manual tracing	Konopaske et al. (2014)
Dendritic spine quantification	Micro/Nanoscale	CM	Spine density, number, and area	Manual tracing	MacDonald et al. (2017)
Functional connectivity	Macroscale	FM	Cell density and FIM	Seg.: stereology–Visiopharm software, semi-automatic FIM: Zen 2.0 Blue Imaging software	Stachowiak et al. (2017)
Structural and functional brain network abnormalities	Macroscale	MRI	Volumes	Prep.,^1^ Seg: surface-based mapping	Bernhardt et al. (2013)
Structural and functional brain network abnormalities	Macroscale	MRI	Volumes	Seg: histology-based volumetry	Bartolomei et al. (2005)
Structural and functional brain network abnormalities	Macroscale	MRI	Volumes	Prep.,^1^ seg: voxel-based volumetry	Bernasconi et al. (2004)
Structural and functional brain network abnormalities	Macroscale	MRI	Volumes and cortical thickness	Prep.,^1^ seg: semi-automatic, surface-based	Bernhardt et al. (2012)
Structural and functional brain network abnormalities	Macroscale	MRI	Volumes and cortical thickness	Seg: surface-based mapping–FreeSurfer v5.3.0	Whelan et al. (2018)
Structural and functional brain network abnormalities	Macroscale	MRI	FA, MD, AD and RD	Prep.,^1^ seg: tensor estimation and tractography	Hatton et al. (2020)
Structural and functional brain network abnormalities	Macroscale	MRI	Structural connectivity	Prep.,^1^ seg.: diffusion tensor calculation and structural connectivity–FDT toolbox	Bonilha et al. (2013)
Structural and functional brain network abnormalities	Mesoscale	CM	Colocalization of immunoreactivity	Manual counting: Adobe Photoshop CS6	Du et al. (2017)
Structural and functional brain network abnormalities	Macroscale	MRI	Functional connectivity	Prep.,^1^ seg: manual and automatic segmentation–AAL	Hong et al. (2019)
Abnormal neuron morphology	Mesoscale	FM, CM, MRI	Cell density, *ex-vivo* FA	Prep.: Image reconstruction–Imaris. Cell seg.: auto-thresholding–ImageJ. DTI seg.: not detailed.	Hsieh et al. (2016)
Abnormal neuron morphology	Mesoscale	CM	Cell density, Sholl analysis, dendritic spine morphology	Prep.: gray-scale conversion, Seg.: Manual cell counting, optical density, Sholl analysis: ImageJ. Dendritic spine: Imaris FilamentTracer module	Wu et al. (2022)
Abnormal neuron morphology	Mesoscale	CM	Cell morphology and density	Seg.: semi-automatic quantification–Analyze Particles on ImageJ and Imaris	Avansini et al. (2022)
Abnormal neuron morphology	Mesoscale	Synchrotron x-ray CT	Cell morphology and density	Prep.: noise reduction. Seg.: threshold, morphological filters and manual correction–Avizo software	Fonseca et al. (2018)
Abnormal neuronal localization	Multiscale:Meso (CM) and Micro: EM	CM, EM	Cell and dendritic spine density and morphology	Prep.: image and neuron reconstruction–Neurolucida. Seg.: automatic morphometry–L-measure. Dendritic spines: manual tracing on SynPAnal. Puncta analysis: ImageJ	Martineau et al. (2018)
Abnormal neuron morphology	Multiscale: macro (RM) and meso (LM)	MRI, LM	Type and number of lesions	Qualitative visual analysis	Dubeau et al. (1995)
Abnormal neuronal localization	Mesoscale	LM	Cell morphology	Qualitative analysis	Ekşioğlu et al. (1996)
Abnormal neuronal localization	Microscale	LM	Cell density	Manual cell counting	Mello et al. (1993)
Abnormal neuronal localization	Microscale	LM, EM	Densitometry, cell morphology	Manual densitometry–Ziess IBAS image analysis system	Babb (1991)

18F-DOPA, 18F-Fluoro-L-Phenylalanine tracer; AAL, automatic anatomic labeling; AD, MD, RD, axial, mean, and radial diffusivity, respectively; BM, brightfield microscopy; CM, confocal microscopy; CONN, functional connectivity toolbox; CT, computed tomography; DAPI, 4′,6-diamidino-2-phenylindole; DMN, default mode network; DTI, diffusion tensor imaging; EM, electron microscopy; FA, fractional anisotropy; FDT, FMRIB’s Library’s Diffusion Toolbox; FM, fluorescence microscopy; FIM, Fluorescence Intensity Measurements; GABA/GABAAR: γ-aminobutyric acid/GABA α-subunit receptor; Glu, glutamate; GM, gray matter; LM, light microscopy; MRI, magnetic resonance imaging; NMDAR, *N*-Methyl-D-aspartate receptor; ROI, region-of-interest; PET, positron emission tomography; Seg., segmentation; Prep., preprocessing; PSI, seed-based d Mapping with Permutation of Subject Images toolkit; T1WI, T1-weighted image; WM, white matter.

^1^Reported preprocessing steps for neuroimaging: slice-timing, attenuation, and motion corrections, registration to T1WI, normalization to neuroanatomical atlas, field-map correction, and smoothing (for functional MRI); intensity correction, registration to neuroanatomic atlas, smoothing; eddy current and susceptibility artifacts correction (diffusion MRI); realignment, motion correction, PET registration to T1WI, normalization to neuroanatomical atlas (for PET).

To gain more understanding of the etiology of these disorders, the integration of morphological and functional 3D data at mesoscale resolution is imperative. Multimodal imaging techniques, including confocal microscopy, light-sheet microscopy, EM, and x-ray tomography, present promising opportunities to obtain a more comprehensive perspective on alterations in neural connectivity. Nevertheless, it is essential to recognize the impracticality of performing live imaging at a mesoscale level in human subjects. In this scenario, robust *in vitro* models, such as 2D neuronal cultures and 3D neural organoid cultures, play a critical role in investigating the complexities of human aberrant connectivity within a controlled environment in a model that more closely resembles human brain development. These combined efforts have the potential to enhance our comprehension of the origins and establishment of aberrant connectivity, and may ultimately contribute to the development of innovative therapeutic approaches.

In recent years, significant advancements have been achieved in the field of mesoscale multimodal imaging, enabling the integration of diverse techniques for comprehensive analysis. Notably, it is now possible to merge a myriad of imaging modalities, resulting in the complete 3D morphological reconstruction of individual neurons while simultaneously acquiring invaluable functional data in view to study global connectivity (Keller and Ahrens, 2015; Kuan et al., 2020; Santuy et al., 2020; Muñoz-Castañeda et al., 2021; Walsh et al., 2021; Bosch et al., 2022; Pisano et al., 2022). Among these techniques are Genetically Encoded Calcium Indicators (GECIs) (Miyawaki et al., 1997; Nakai et al., 2001), with the recently developed CaMPARI (calcium-modulated photoactivatable ratiometric integrator) emerging as a notable standout in mesoscale imaging (Fosque et al., 2015). CaMPARI distinguishes itself by its unique feature of irreversibly labeling photoconverted neurons, extending the observation of active networks beyond the initial snapshot of activity. This capability has been leveraged to capture task-dependent activity patterns across brain regions and visualize hippocampal synaptic plasticity in freely moving animals (Berndt et al., 2023; Das et al., 2023). Notably, the practicality of CaMPARI is enhanced by its capability for multiple uses in longitudinal *in vivo* studies (Das et al., 2023). Furthermore, the single-cell precision of CaMPARI facilitates the exploration of interconnected microcircuits, allowing for the evaluation of disruptions in excitatory and inhibitory (E/I) signaling (Martin and Plavicki, 2020), a crucial factor in connectivity influencing conditions such as schizophrenia and epilepsy. This remarkable progress reflects the convergence of innovative technologies and methodologies, leading to a deeper understanding of neural structures and their structural and functional connections at the mesoscale level.

In this context, there are several gaps in understanding disorders affected by brain connectivity that could be addressed by leveraging mesoscale-related approaches. In schizophrenia, delayed PFC maturation, specifically GABAergic interneurons, contributes to cognitive and social deficits in adolescence (Lewis, 1997; Caballero and Tseng, 2016; Delevich et al., 2018). Investigating prefrontal circuitry formation and the impact of excitatory inputs from subcortical regions on interneurons vs. pyramidal neurons in the PFC is crucial. CaMPARI, for example, could offer valuable means to investigate these dynamics. Integrating 3D models with mesoscale imaging (e.g., confocal or live cell imaging and functional calcium imaging) can address these questions, revealing dynamic processes and synaptic development in the neuronal circuitry.

Likewise, in epilepsy research, we may inquire about the processes involved in the conversion of a focal seizure into a generalized event encompassing several cortical areas by addressing questions such as: What factors drive this electrical propagation? Is it the result of abnormal neurite branching patterns or an unusual number of dendritic spines? Moreover, it remains imperative to determine the specific neural cell type responsible for orchestrating the shift from a localized circuit, synchronizing neighboring cells, to the initiation of a generalized ictal event. Thus, studying brain network development and organization in the mesoscale will allow us to understand seizure formation and spread.

## 3 Image processing: quantifying connectivity

**FIGURE 2:**
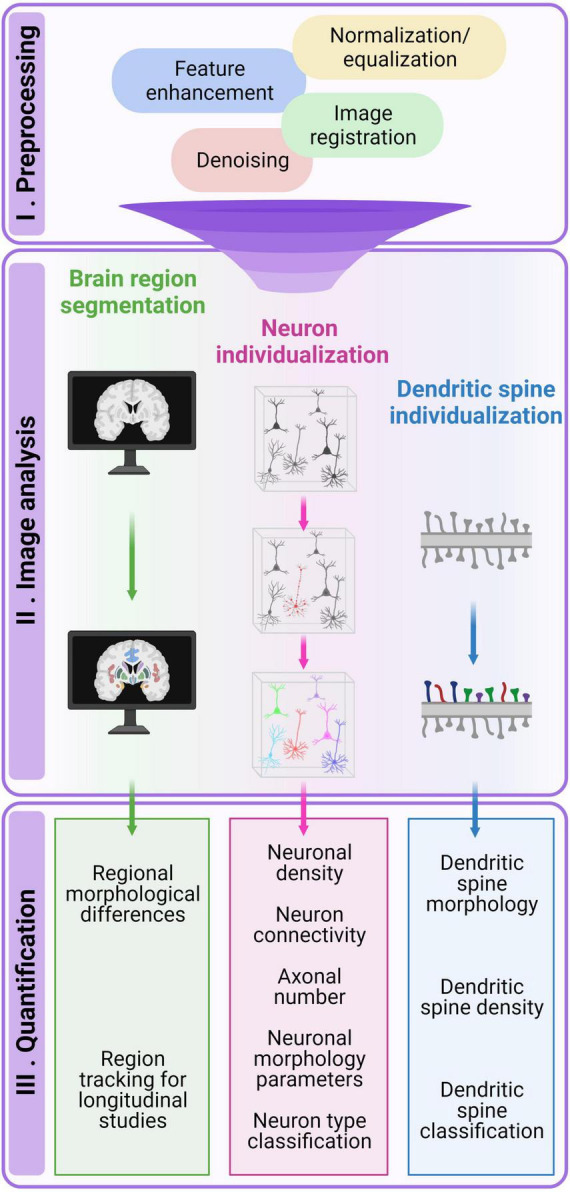
Overview of the bioimage analysis workflow. The pipeline generally comprises three fundamental steps: image preprocessing (I), image analysis (II), and quantification (III). In the preprocessing step, raw image quality is enhanced to facilitate subsequent analysis or visualization. Image analysis involves identifying and delineating specific regions or objects of interest, which is essential for extracting quantitative data from bioimages. The quantification step involves extracting meaningful quantitative measurements and deriving insights from the acquired images.

Image processing tools are essential for quantifying data and revealing the intricate relationships between brain networks and aberrant connectivity. Image processing techniques can extract qualitative and quantitative measurements from a variety of neuroimaging modalities, including MRI, two-photon, confocal, super-resolution, microscopy, and EM. Initial steps involve the identification of which information the research needs to extract from the data (e.g., tiny structures), followed by the selection of algorithms and their fine-tuning on a particular data (e.g., noise filtering, contrast enhancement). After establishing an adequate workflow, the outcome must be validated by expert neurobiologists. During this stage of image processing, human input on several levels inevitably leads to undesired bias or even difficulties in identifying subtle information such as fine morphological structures. Adding to this equation, the amount of raw data is sometimes not feasible to be fully accomplished manually, and this is especially true for mesoscale generated data. In this scenario, the development of automated or semi-automated computerized processing is paramount to achieving an efficient large-scale data processing. In general, a typical processing workflow consists of three fundamental steps: image preprocessing, image analysis, and quantification (Figure 2). However, it is important to note that specific modifications on the pipeline are required based on the type of image used and the particular neural structure under investigation. While a general image analysis pipeline can find utility in various scenarios, it is important to recognize that each biological question has a unique demand, and this requires the development of dedicated processing pipelines.

In the following section, we will explore the most suitable image acquisition and processing techniques for tackling key issues associated with conditions affected by abnormal brain connectivity. These issues encompass inter-regions brain connectivity, axonal and soma density, single neuron morphology, and dendritic spine quantification and morphology. Our approach will commence with the macroscopic analysis of brain regional images and end with the micro-scale assessment of dendritic spine quantification and morphology. We will not address image contrast enhancement and noise reduction preprocessing strategies as they have several computational implementations in each of the acquisition techniques and it could divert our focus from the main goal of this section: understanding the crucial role of segmentation and data analysis in comprehending connectivity. For an in-depth review of image denoising, the interested reader is referred to Kollem et al. (2019), Kaur et al. (2021), and Huang C. et al. (2022). There is a plethora of manual image analysis tools but in this review we will focus on automatic or semi-automatic quantification.

### 3.1 Quantifying inter-regional brain connectomics

Anatomically, the brain is compartmentalized into distinct regions, each with designated functions that collectively contribute to a range of high-order cognitive processes. Inter-regional brain connectomics consists of mapping and studying the complex networks between different regions (Behrens and Sporns, 2012). By analyzing these connections, using either macro, meso, or multiscale imaging strategies, it is possible to gain insights into how different regions cooperate or compete (Behrens and Sporns, 2012), and how disruptions in these networks may lead to neurological or psychiatric disorders. The macroscale approach focuses on imaging techniques that encompass the entire brain, ideally *in vivo*, with a selection of modalities such as MRI for structural covariance, fMRI, diffusion weighted image (DWI–including DTI and tractography), and PET. Structural connectivity, primarily addressed through DWI/DTI and tractography, when combined with fMRI, can also reveal structural connectivity (Axer and Amunts, 2022).

In the context of computational processing for macroscale images, artificial intelligence methods for image analysis are widely utilized in diagnosis contexts to understand neurological and psychological disorders (Zhang et al., 2020). For meso and micro scales, image analysis techniques currently available to extract neural connectivity lie within the segmentation and neuron individualization. Pixel/voxel classification, frequently called Region of Interest (ROI) delimitation, is the first step to a multitude of tasks. Once an ROI is defined, it becomes possible to trace morphological markers in longitudinal or comparative studies. Furthermore, it aids in the precise delineation of abnormal regions, guiding surgeons in tasks such as tumor extraction or identifying the epileptogenic zone by PET/fMRI images. In longitudinal developmental studies, segmenting regions like the prefrontal cortex over time provides valuable insights into the maturation of connectivity networks associated with cognitive development (Liu et al., 2023). ROI segmentation is also a crucial step for morphological quantification assessment as it enables researchers to access region volume or identify morphological differences in patients compared to control individuals in MRI.

Within psychiatric imaging, schizophrenia research has consistently revealed two prominent observations: increased cerebroventricular size and reductions in cerebral gray matter volume (Ananth et al., 2002; Shapleske et al., 2002). Automatic ROI segmentation and morphometric quantification of gray matter volume in MRI images decrease human biases and help to evaluate different groups in comparative or longitudinal studies (Fornito et al., 2017; Nemoto et al., 2020). While traditional image processing techniques such as thresholding-based segmentation, watershed labeling, neuroanatomical-atlas-based segmentation, or semi-manual masking [using tools like FreeSurfer (Fischl, 2012) or BET (Smith, 2002) are available, the medical context often requires greater accuracy even on images with unclear borders or blurred definition (Wang et al., 2023)]. In this context, several machine learning techniques have been successfully used in analysis of complex datasets, including k-means clustering, Support Vector Machines (SVM), Random Forest, Adaptive Boosting (AdaBoost), eXtreme Gradient Boosting (XGBoost) and Deep Learning strategies like Convolutional Neural Networks (CNN), Generative Adversarial Networks (GAN), Recurrent Neural Networks (RNN) (Wang et al., 2014; Zhang Z. et al., 2021; Verma et al., 2023).

In the field of epilepsy, image segmentation or ROI delimitation primarily aims to locate the epileptogenic zone and define pre-operative surgical areas. While this kind of analysis is commonly applied to MRI-T1 and fMRI images (Segato et al., 2020), its applicability extends to PET, DTI, and DWI scans (Sollee et al., 2022). For instance, in the study conducted by Lee et al. (2020), the authors used deep-learning CNN to pinpoint specific regions for surgical resection in DWI and tractography images of pediatric patients. Additionally, (Zhang Q. et al., 2021) constructed a pair-of-cube (PoC)-based Siamese CNN using two identical 18-layer ResNet to identify epileptic focus in F-fluorodeoxyglucose (F-FDG) PET images. After localization, the metabolic abnormality level of the predicted focus was automatically determined using the asymmetric index (AI). In another instance (Li K. et al., 2019; Vakharia et al., 2019) conducted detailed segmentation of critical areas, including the ventricular system, brainstem, amygdalohippocampal complex, parahippocampal gyrus, and sulci, from MRI-T1 9 images. Subsequently, they employed Random Forest algorithms to preplan laser trajectories of respective surgeries of epileptic zones with less adverse events associated with epilepsy surgery. For a comprehensive exploration of how deep learning techniques can be used in epilepsy, we recommend Sollee et al. (2022) review.

### 3.2 Multiscale imaging: bridging micro to macroscale

Macroscale inter-regional insights are directly associated with microscale synaptic organization and arborization (Wei et al., 2019). The overall cortico-cortical connectivity observed at the macroscale in BigBrain profiles is strongly correlated to microscale laminar cytoarchitectonic patterns (Wei et al., 2019). Essentially, cortical regions exhibiting higher similarity in microscale patterns are more likely to be interconnected (Wei et al., 2019).

Additionally, multiscale approaches, which integrate data from various imaging modalities, hold the potential to interlink micro and macro scales. For example, the BigMac dataset, developed by Howard et al. (2023), combines *in vivo* MRI images with post-mortem microscopy data and ultra-high angular resolution diffusion imaging and enables the mapping of microscale cellular structures to macroscale features. This comprehensive approach allows researchers to study brain connections at both macro and micro levels, bridging the gap between them.

However, Haueis (2021) cautioned against oversimplifying the micro-to-macro correlation by merely averaging microscale details. Failing to account for the intermediate mesoscale structure and organization in this practice may lead to analytical errors. Haueis further emphasized the critical role of mesoscale circuit organization in accurately depicting the structure-function relationship, particularly in the context of cortical gradient modeling. This is a compelling piece of evidence that bridging micro-to-macro scale connectivity should pass through mesoscale circuit understanding.

### 3.3 Mesoscale imaging

The trade-off between image resolution and sample size in 3D is a well-known limitation. The higher spatial resolution comes at the cost of a smaller field of view (FOV). Nonetheless, mesoscale brain imaging strategies combine cellular-level resolution and an extended spatial range. The primary approach employed in mesoscale imaging involves the use of wide-field or laser-scanning confocal microscopies, heavily impacted by the thickness of the sample. Recent methodologies such as light-sheet and two-photon partially overcome this limitation by going deeper inside intact tissues, while preserving high spatial resolution (Cazemier et al., 2016; Tyson and Margrie, 2022). For example, Li et al. (2010) used an automatic micro-optical sectioning tomography (MOST) to obtain a mesoscale atlas of the mouse brain. This strategy integrates a microtome, light microscope, and image recorder, and allows for simultaneous imaging and sectioning (Li et al., 2010). Another possible approach was the use of post-mortem axonal projections enhanced by green fluorescent protein (EGFP)-labeling (Oh et al., 2014). They imaged many small patches of brain tissue with two-photon microscopy to form a big image with cellular-level resolution (Oh et al., 2014). Imaging at this scale in larger FOV took 18.5 h of scanning and resulted in a 750 GB raw dataset. Likewise, (Wang et al., 2019) developed the VISoR system, a sophisticated adaptation of light sheet microscopy, to obtain 3D mouse brain images with neurite resolution within 1.5 h.

**FIGURE 3:**
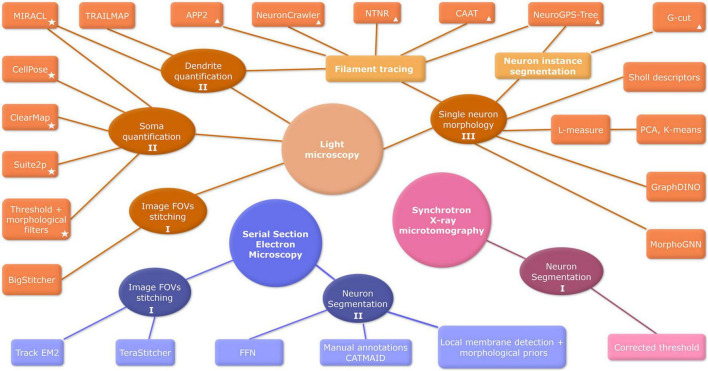
Overview of mesoscale bioimage processing methods. Image acquisition techniques are represented by big circles: Light Microscopy (orange), Serial Section Electron Microscopy (blue), and Synchrotron X-ray (pink). Image processing steps are depicted by ellipses, and algorithms of analysis are represented by boxes. Labels I, II, and III denote the fundamental steps of mesoscale image analysis: preprocessing, segmentation, and data analysis, respectively. Algorithms tools are referenced in Table 2 (✩) and Table 3 (Δ). FOV, field of view; FFN, flood-filling network; CNN, convolutional neural network; EM, electron microscopy; PCA, principal component analysis.

Another time-optimizing approach for mesoscale involves the use of synchrotron X-ray imaging. Especially in 3D computed tomography is becoming popular since the higher energies of x-rays allow deeper penetration and very high resolution. Although it can take a few hours to measure a sample in benchtop equipment, synchrotron sources emerge as a solution for fast measurements and even higher spatial and temporal resolutions, which also allow a combination of several tomograms to reconstitute large FOV (Fonseca et al., 2018; Rodrigues et al., 2021; Claro et al., 2023). Image processing pipelines are usually developed for a specific imaging acquisition technique. A comprehensive summary of primary mesoscale image processing methods for the main image acquisition modalities can be found in Figure 3.

#### 3.3.1 Processing at the mesoscale level: insights into neurite and soma regional density

Extensively imaging and tracing axons throughout the brain provides a mesoscale view of regional connectivity, offering insights into soma and neurite density as well as assessing total cell reductions and identifying cell death in specific brain regions (Bazinet et al., 2023). Although mesoscale imaging strategies can unveil a series of histological structures, they present their computational challenges.

The first challenge encountered in the mesoscale is usually the stitching of large quantities of high-resolution microscopy images. Image stitching refers to the process of aligning and overlaying two or more images of the same object taken from different, consecutive, and overlapping FOV. Through image registration processing (Sarvaiya et al., 2009), corresponding features or structures in these images are spatially aligned, making it possible to combine them into a single and panoramic image. In the case of brain microscopy, this involves merging multiple images, sometimes acquired at varying scales, into a comprehensive, high-resolution representation of the brain. Registering can mean either tile stitching multiple consecutive FOVs, without overlapping or positioning microscopic images into a larger dataset using fiducial markers, or a common reference frame to localize them into the brain. In both cases, it is expected that mesoscale imaging strategies generate the largest amount of raw data. BigStitcher is a method of stitching consecutive FOVs into a single high-resolution image (Hörl et al., 2019). To manage such large amounts of data, the BigSticher software computes shifts between overlapping image tiles by using a phase correlation method in downsampled images, which optimizes the computational time necessary for image stitching (Hörl et al., 2019). Moreover, DeepSlice is a CNN specifically trained on a substantial histological dataset to automatically align coronal mouse brain two-photon microscopy images with the Allen Common Coordinate Framework (CCF) (Carey et al., 2023).

The following step is to detect neuronal cell bodies in the entire image and quantify soma density across brain regions. The size of mesoscale data makes manual handling impractical and prone to biases; hence, automatic or semi-automatic tools are more suitable for its processing (Bjerke et al., 2023). Soma detection can be made either by traditional image enhancement filters followed by intensity thresholds, such as in ClearMap (Renier et al., 2016) and MIRACL (Goubran et al., 2019), or by advanced machine learning techniques for pixel classification as deep learning approaches (Tyson and Margrie, 2022). Intensity thresholding approaches also work well with DAPI-stained nuclei images (Kim et al., 2015), and modifications of thresholding can be done to address large-scale GFP images even with a large variability in contrast (Frasconi et al., 2014). These modifications consist of first using mean shift clustering to detect soma centers followed by image deconvolution and finally manifold learning for filtering false positives (Frasconi et al., 2014). However, intensity thresholding and morphological approaches fail especially with densely packed images and that is precisely where deep learning can be used. For example, (Hu et al., 2021) combined 3D U-shaped full CNN with multi-task learning to perform soma segmentation in Nissl stained images. This strategy is done in small patches and would take a long time to train in teravoxel mesoscale images. As a faster approach, (Wei et al., 2023) used a lightweight MCC-Net to reduce computational complexity in soma detection. Then, in the second stage, they employed SFS-Net for precise soma localization in mouse brain images, utilizing advanced segmentation techniques. Experimental results confirmed the excellent performance of the method and its capacity to provide valuable information for neuron reconstruction (Wei et al., 2023). The user-friendly software CellPose (Stringer et al., 2021) also has a DeepLearning module that has been used to count pyramidal neurons in histopathological images (Oltmer et al., 2023). An alternative option is to employ Suite2p software (Pachitariu et al., 2017), which offers AutoROI cell segmentation designed for simultaneous analysis of functional and morphological two-photon calcium images. The compilation of the main soma quantification methods for mesoscale connectivity is presented in Table 2.

**TABLE 2:** Main soma quantification methods for mesoscale connectivity.

Soma quantification method	Sample preparation/microscopy	Principle
Kim et al. (2015)	DAPI stained nuclei	Intensity threshold
Frasconi et al. (2014)	GFP transgenic mice	Adaptation of intensity threshold: mean shift clustering to detect soma centers, supervised semantic deconvolution by means of neural networks for image enhancement and manifold learning for filtering false positives
ClearMap	YFP	Nuclei detection with background subtraction, filters, morphological operations, and 3D peak detection, followed by watershed segmentation and volume-based filtering to identify cells.
MIRACL Pipeline	YFP + DTI registering	Segmentation workflow in ImageJ, utilizing optimized pre-processing, morphological analysis algorithms, and a parallelized feature extraction algorithm for 3D cellular features.
CellPose (Stringer et al., 2021; Oltmer et al., 2023)	Light microscopy, HE stained histopathological images	A simulated diffusion process generates spatial gradients pointing toward the center of a cell, and a neural network trained on these gradients, along with pixel categorization, forms a gradient vector field used to predict masks by constructing a dynamical system with fixed points.
Suite2p (Pachitariu et al., 2017)	Two-photon calcium images	Greedy segmentation of nearby pixels
Hu et al. (2021)	Nissl stained	Modified 3D fully connected Unet
Wei et al. (2023)	fMOST	Lightweight neural network for quick soma detection in low resolution, followed by a network with multi-scale context and a module for precise soma localization.

DAPI, 4′,6-diamidino-2-phenylindole; GFP, green fluorescent protein; YFP, yellow fluorescent protein; DTI, diffusion tensor imaging; fMOST, fluorescence micro-optical sectioning tomography; 3D, three-dimensional; HE, Hematoxylin and eosin stain.

The next step to a mesoscale connectivity view is to detect, trace, and quantify neurites across the brain. In the study conducted by Allen Mouse Brain Connectivity Atlas (Oh et al., 2014), axonal detection at the mesoscale level starts with a very similar process to single neuron morphology mesh tracing. Signal detection algorithms, such as filament tracing, can be used as an initial segmentation. The authors then rescale image intensity and remove noise using filters and morphological techniques. Candidate signal objects were identified based on adaptive edge/line detection and morphological attributes such as length and area. Additionally, high-intensity pixels near these objects were considered. In a post-segmentation step, objects considered artifacts were removed. It is important to note that passing fibers and terminals were not differentiated. The outcome is a high-resolution mask classifying each 0.35 μm × 0.35 μm pixel as a signal or background (Oh et al., 2014).

Also in the mesoscale, TRAILMAP uses a modification of a 3D UNet to extract axonal projections from uncleared brain tissue in light-sheet microscopy (Friedmann et al., 2020). This method focuses on segmenting axons from the background in a generalized way that can be applied to all brain regions. Unlike filament tracing methods, it does not address neurite branching numbers or spatial positions. The purpose is mainly to address axonal fiber density and compare it across brain regions (Tyson and Margrie, 2022).

Another possibility is to use the MIRACL pipeline and toolbox. MIRACL is based on a multimodal approach that integrates CLARITY data at the microscopic level with macroscopic *in vivo* and *ex vivo* imaging data, including structural, diffusion, and quantitative MRI, all aligned to the Allen atlas reference frame “ARA.” This integration facilitates various analyses, including the examination of histological features across network graphs and fiber tracts, as well as connectivity analyses based on projection terminals. Additionally, MIRACL supports group-level statistics, multimodal correlations, and comparisons of connectivity maps across different scales (Goubran et al., 2019).

#### 3.3.2 Single neuron morphology

The morphology of a neuron can have a big impact on its connectivity with other local neuronal circuits. Neurons with a complex dendritic branching pattern tend to have a larger surface area and a denser synaptic field, allowing them to have more candidate synapses (van Pelt and van Ooyen, 2013). According to Peter’s rule, the colocalization of dendritic and axonal arbors are reasonable predictors of connectivity among neuron types (Rees et al., 2017). Nevertheless, a greater number of potential synapses does not always mean a greater number of functional synapses (Rees et al., 2017). Axo-dendritic overlapping is a necessary but not sufficient condition to ensure a synaptic connection. Light microscopy is the ideal imaging technique to study both neuronal morphology (using cytoplasmic markers) and synaptic connectivity (using puncta colocalization) (Wang et al., 2020). In contrast, neuron morphology has recently been described to predict non-random connectivity in local networks and circuits (Udvary et al., 2022). The authors state that the specificity in neural wiring is influenced by morphological factors such as similarities in neurite projections, packing density, and the diversity of cell types in the neuropil (Udvary et al., 2022). High values in these factors lead to recurring patterns in the network, while lower values result in a more feedforward network structure (Udvary et al., 2022).

To address single neuron morphology using imaging techniques we must first extract from the image which pixels belong to each neuron. This process is called instance segmentation or neuron instance individualization (You et al., 2019). The main techniques used to measure multiple neurons are light microscopy (including confocal, two-photon, STED, and light-sheet) and serial-section EM.

##### 3.3.2.1 Single neuron morphology using light microscopy

Traditionally in light microscopy (confocal, light sheet, and STED), neuron instance individualization starts with filament tracing methods (Xiao and Peng, 2013; Feng et al., 2015; Liu et al., 2016; Quan et al., 2016; Shih et al., 2021). These methods work as an initial segmentation and are responsible for differentiating the neural mass foreground from the noisy scattered background (Magliaro et al., 2019). They transform an image into a graph of connected points. Filament tracing pipelines consist mainly of four steps: (i) an image pre-processing step to improve signal-to-noise ratio enhancing filaments and smoothing background; (ii) a seed point detection step followed by (iii) “energy minimization algorithms” such as Fast Marching Method (FMM) (Sethian, 1996) and Dijkstra algorithm (Dijkstra, 1959); and (iv) a pruning step to reduce redundant traces and improve overall segmentation (Liu Y. et al., 2022).

In the pre-processing step, the main goal is to significantly enhance the signal-to-noise ratio. In confocal imaging techniques, the pre-processing involves PSF (point spread function) deconvolution, feature-enhancing filters (Frangi et al., 1998), or deep learning techniques that enhance neurons based on a predicted morphology (Yang et al., 2021a). Then, seed point detection usually includes the detection of somata searching for the brightest point on the image (Xiao and Peng, 2013). After that, energy minimization algorithms find the shortest path between a starting point and all other points in a graph, using a cost function usually based on image intensity or transformed distance of a filament. The final step is filament pruning and morphological corrections (Liu Y. et al., 2022). These traditional tracing methods are very robust and widely used in neuron imaging. Nonetheless, most algorithms are not optimized for large volumetric images (giga or tera voxels) and images with densely packed cells. If the traced image contains multiple neurons this will result in a mesh containing all cells and will require further neuron individualization.

Nonetheless, filament tracing has improved a lot since the 2008’s DIADEM golden age. The Big Neuron Project and Mouse Light Project have reconstructed over 1000 neurons and are constructing a database. Neuron Crawler (Zhou et al., 2015) has begun solving the big data problem and, Deep Learning methods have been helping to improve the tracing framework (Dai et al., 2019; Tan et al., 2020; Huang et al., 2021; Yang et al., 2021b; Liu C. et al., 2022; Wang et al., 2022). Table 3 summarizes the main filament tracing methods used in the mesoscale connectivity.

**TABLE 3:** Main filament tracing and neuron individualization methods for mesoscale connectivity.

Method	Microscopy	Overview	Sparse/dense	References
APP2	Confocal	Image enhancement step followed by seed point detection on local maxima and FFM	Originally created for single neuron use. But can be used on sparse images	Xiao and Peng (2013)
NeuronCrawler	Confocal	Similar to APP2 but improved to large images	Originally created for single neuron use	Zhou et al. (2015)
CAAT	fMOST	3D CNN predicts object probability, followed by an adaptive voxel scooping approach on the probability map,	Dense and large scale neuron tracing	Huang et al. (2021)
NTNR	Ultra-scale optical microscopy	A hybrid model. CNN backbone merged with a Transformer encoder-decoder architecture	Dense	Wang et al. (2022)
G-Cut	Confocal	Requires prior tracing and soma identification. Somas are used as seeds and adaptation of Djikstra’s algorithm based on morphological priors is used to segment neuron instances	Dense	Li R. et al. (2019)
Neuro-GPS-Tree	Many modalities	Uses local and global cues to automatically classify neurites and reconstruct large-scale neuronal populations with dense neurites	Dense	Quan et al. (2016)

FFM, fast marching method; fMOST, fluorescence micro optical sectioning tomography. Adapted from Magliaro et al. (2019).

Once the neuronal mesh is accurately traced by filament tracing strategies, the next step is to individualize each neuron. Algorithms such as G-Cut (Li R. et al., 2019) and NeuroGPS-Tree (Quan et al., 2016) use the soma identity and position, and from previously learned morphological parameters trace the most probable neuron given the soma and the traced neuronal mesh.

##### 3.3.2.2 Single neuron morphology using electron microscopy

Electron Microscopy can also uncover single neuron morphology and local connectomics with nanometric synapse level resolution. Using EM to reconstruct neuron wiring and connectivity involves multiple steps: high-throughput data acquisition, image registration, image segmentation, proofreading, and tracking (Beyer et al., 2022). Unlike confocal or light sheet microscopy, 3D EM neuron reconstruction requires physical sectioning of the sample. The samples are cut into about 30 nm thick samples and individually imaged. A 1 mm^3^ brain sample requires about 5000 slices, 2.1 petabytes of raw microscopy data, and 326 days to finish data acquisition (Shapson-Coe et al., 2021). Similarly, (Winding et al., 2023) imaged 3016 neurons and 548,000 synapses in a Drosophila larval brain. The resulting image contained 4841 z-slices and processing it took manual annotation of multiple users and a specialized annotation tool for big images (CATMAID) (Winding et al., 2023).

After each image has been acquired the next step is to stitch adjacent 2D images and correctly stack (register) them to form a 3D volume. During 3D thin-sliced EM image acquisition, the most fundamental step for proper 3D reconstruction is image registration. Aligning microscopy slices can be challenging since they are not perfectly aligned and often have different quality and acquisition parameters (Beyer et al., 2022). The main 2D stitching includes plugins such as TeraSticher (Bria and Iannello, 2012) and 3D registration can be done with the ImageJ plugin TrackEM2 (Cardona et al., 2012). Once the image volume is completed, the next step is to individualize and segment each neuron. Due to the highly textured nature of EM images, segmentation is typically accomplished using deep learning techniques (Shapson-Coe et al., 2021), using a flood-filling network (FFN). Most of the EM segmentation algorithms rely on detecting cell membranes to separate neurons, and even small errors in this detection could split or merge neurons, significantly impacting the reconstructed neural circuit (Krasowski et al., 2018). In this context, combining neuro-morphological priors with local membrane information can be a viable resource to reduce errors in the neuronal individualization process (Krasowski et al., 2018; Hong et al., 2023).

#### 3.3.3 Morphology quantification of individual neurons

The first and most important parameter to quantify single neuron morphology is the radial profile of neuron dendrite spanning tree, also known as the Sholl Intersection Profile (SIP) (Bird and Cuntz, 2019). The complete Sholl analysis includes measuring the total length of the dendrite, the axon domain maximum and minimum from the soma, and the angular distribution of dendritic segments that deviate from a direct path to the soma. According to the authors (Bird and Cuntz, 2019), a larger dendrite extension length implies a larger region where synapses can occur, peaks in the SIPs are related to regions where synapses have a higher probability to occur and valleys in the SIPs are regions to where synapses have a lower probability to occur. The angular distribution is related to a neuron’s centripetal bias and implies a neuron that minimizes wiring to ensure an efficient propagation of electrical impulses.

Alternative ways to measure neuronal shape include parameters such as the total length of neurites, the minimal occupied volume, the distribution of branch lengths as represented in histograms, and the frequency of distances between successive bifurcations along the neural trajectory. These measurements are obtained through the open-source software L-measure, as outlined in the work of Scorcioni et al. (2008).

A further challenge is to classify neuron types using only their morphological assets without any molecular markers (Polavaram et al., 2014) used L-measure to extract morphological features of neurons in the NeuroMorpho database. They subsequently applied principal component analysis (PCA) as a statistical tool to identify key morphological parameters capable of effectively classifying dendritic structures across diverse metadata categories. Their findings highlight the importance of specific measures like branching density, size, tortuosity, bifurcation angles, arbor flatness, and topological asymmetry in capturing meaningful features of dendritic trees. Similarly, Khalil et al. (2021) extracted L-measure metrics and modified Sholl descriptors from the NeuroMorpho database and used PCA and KNN clustering to classify neuronal types.

Deep learning revolutionized feature extraction and image classification and has been used to classify neurons. For example, GraphDINO used a Transformer-based Graph Neural Network to create 3D spatial embedding representations of neuronal graphs and later classified them into neuronal types (Weis et al., 2021). The authors adapted positional encoding and introduced a novel attention mechanism called AC-Attention to fit neuronal graphs and achieved results comparable to expert-manual classification without prior knowledge about neuronal structural features and outperforms previous methods in predicting expert labels on quantitative benchmarks (Weis et al., 2021). Similarly, MorphoGNN is a novel approach for embedding single neuron morphologies using graph neural networks (GNN) and learns spatial relationships between nodes in reconstructed neuron fibers by considering their nearest neighbors on each layer. This process generates a reduced-dimensional representation of individual neurons using an end-to-end model that incorporates densely connected Densely Connected Convolutional layers and a dual pooling operator (Zhu et al., 2023).

### 3.4 Dendritic spine quantification and morphology

Dendritic spines are small protrusions from dendrites that constitute the center of excitatory synaptic interaction among central neurons (Papa et al., 1995). They are crucial structures for interneuronal communication and play a crucial role in learning and memory. Neuronal spines can range in size from tiny, barely visible protrusions to larger and more complex structures. This variety suggests that neuronal spines have a wide range of functions and are essential for neural plasticity and cognitive and sensory functions (Rochefort and Konnerth, 2012; Ekaterina et al., 2023).

The analytical approach is often used to study dendritic spines, including their density and respective morphological features (Chang et al., 2017). Light microscopy and EM can image dendritic spines and monitor their dynamic alterations in response to neural network activity (Arellano et al., 2007). In this section, we have chosen to emphasize image processing tools obtained by light microscopy.

Traditionally, dendritic spine images are obtained through Golgi staining and wide-field microscopy. 3D studies of such structures can benefit from confocal reflection imaging, although manual dendrite tracing is still in place. Popular software like Imaris (Govindan et al., 2021), or NeuroLucida (Dickstein et al., 2016), followed by the utilization of semi-automatic measurement tools such as software like SPINEJ (Levet et al., 2020) and NeuronStudio (Rodriguez et al., 2008) have a broad use. To employ deep learning for automated methods, it requires extensive datasets comprising meticulously segmented, high-quality images, known as “*ground truth images*” (Vidaurre-Gallart et al., 2022). However, it’s important to note that even with such datasets, there may still be limitations to achieving precise reconstructions (Vidaurre-Gallart et al., 2022).

The image processing routine for analyzing dendritic spines involves a five-step pipeline: (i) data pre-processing as described before, (ii) spine location detection, (iii) segmentation to isolate them, (iv) quantification of morphological characteristics, and (v) classification or clustering based on their morphology (Li et al., 2023).

The primary objective in the spine detection phase is the precise identification of individual entities’ locations within the 3D image (Rodriguez et al., 2008). This process begins delineating dendrite boundaries, utilizing information extracted from the dendrite 3D mesh (Mukai et al., 2011; Okabe, 2020). There are four main spine detection automatic approaches. The most prevalent method is skeletonization, which involves the removal of consecutive layers of pixels from the dendritic boundary (Okabe, 2020). To detect spines using skeletons, it is necessary to binarize the original images correctly and extract all spines that are still connected to dendritic shafts. If any spines become disconnected during the binarization process, they need to be reattached through further processing (Rusakov and Stewart, 1995). The Rayburst sampling (Rodriguez et al., 2006, 2008), gradient-based methods (Zhang et al., 2010), and analysis of 3D surfaces (Li and Deng, 2012) represent alternative automated approaches for spine detection.

For spike detection, it is necessary to establish the boundary that separates the spines from the dendritic shafts, using iterative methods (Okabe, 2020). One way to perform automatic spine segmentation using light microscopy involves a calculation of the distance to the surface of the neuritic shaft for each voxel outside the shaft (Rodriguez et al., 2008; Singh et al., 2017).

After segmentation, a variety of spine morphological measurements and posterior spine classification can be automatized. Parameters of the 3D structure of spines encompassing spine length, head diameter, neck length, volume, curvature, basal radius, maximum and minimum radius, and head-to-neck ratio (Rodriguez et al., 2006; Janoos et al., 2009). After 3D neuronal morphometry, various principles for spine classification have been proposed and the commonly employed method involves categorizing spines into four main groups stubby, thin, filopodia, and mushroom-shaped (Hering and Sheng, 2001). While traditional phenotypic classification often relies on manual inspection, machine learning approaches, aided by labeled training datasets, have demonstrated comparable accuracy to human operators (Basu et al., 2018), most of them using semi-supervised learning (Shi et al., 2009, 2014). Computational analysis of 3D spine morphology has the potential to unveil novel spine characteristics by fusing clustering methods to automatically group spines with similar structures. Luengo-Sanchez et al. (2018) proposed a probabilistic approach that categorized the spine in clusters based on a selected set of morphology features, with a Gaussian finite mixture model.

The rise of the high-resolution light microscopy image era has led to an expansion of techniques for automated spine detection, segmentation, and measurement. For a comprehensive overview, we recommend a thorough review presented by Okabe (2020).

## 4 Challenges and perspectives

In the examination of mesoscale connectivity within the context of connectivity-related brain disorders, we highlighted the following challenges: (i) refinement of human models; (ii) enhancement of imaging acquisition; and (iii) optimization of computational processing.

Human neural organoids are revolutionizing the study of neural development and diseases in a controlled *in vitro* setting, overcoming the limitations of traditional animal models. These organoids recapitulate the complexities of neural development, offering insights into health and diseases (Avansini et al., 2022). The *in vitro* system allows for drug testing, intervention studies, and close observation of potential side effects. Organoid models support experiments and correlative microscopy in multimodal platforms, enabling comprehensive characterizations of entire samples *in vivo*. This approach represents a significant stride in neurobiology and drug development. Neural tracing using viral vectors and X-ray markers offers precise tools to investigate neural connections and circuitry, enhancing imaging capabilities for detailed visualization and mapping of neural structures. This combination facilitates a deeper understanding of neural development.

From the perspective of image acquisition, EM provides unparalleled spatial resolution at the sub-micron to nanoscale, but it comes with challenges, including difficulties in measuring samples several micrometers thick due to the destructive nature of sample preparation for transmission images and limitations on molecular markers. Photon-based microscopies offer an alternative, capable of imaging multiple cell layers with single-cell identification resolution. Visible light microscopies simultaneously label numerous molecular markers, but a new physical phenomenon limits resolution due to the larger wavelength of light. Super-resolution microscopies (e.g., STED, SIM, PALM/STORM) overcome this limitation and are now widely available in bioimaging facilities, paving the way for enhanced imaging beyond traditional light microscopy constraints.

Expansion microscopy techniques have recently proven effective in reconstructing neuronal connections by employing a water-swellable polymer to expand tissue samples, overcoming optical microscopy limitations (Chen et al., 2015; Gallagher and Zhao, 2021; Lillvis et al., 2022; Kraft et al., 2023). This approach preserves sample integrity while providing detailed insights into cellular and sub-cellular details, including cell projections and connections.

A complementary approach involves increasing photon energy (i.e., shortening the wavelength), with X-rays being a prominent choice due to their deep penetration and high resolution. Although not practical for most benchtop equipment, synchrotron radiation techniques have demonstrated feasibility in neuronal connectomics, offering effective contrast for both unstained (phase propagation) and contrast-enhanced (absorption) samples (Kuan et al., 2020; Rodrigues et al., 2021; Claro et al., 2023). Scanning X-ray fluorescence can map cellular and subcellular chemical elements, potentially providing a biochemical signature for specific disorders (Finnegan et al., 2019; Álvarez-Marimon et al., 2021). Correlative Light and Electron Microscopy (CLEM) is a promising technique that seamlessly combines the advantages of light microscopy, such as molecular markers, with the high spatial resolution of EM. Particularly valuable for studying neural circuits, CLEM generates synaptic-level resolution images across a large field of view, revealing extensive neural circuitry. Its ability to incorporate fluorescent markers streamlines post-processing segmentation, resulting in a more precise reconstruction of neural networks (Iwasaki et al., 2022). It is important to highlight additional aspects of multimodal imaging. APEX2 and MiniSOG serve as genetic tags that are applicable not only in EM as molecular markers but are also suitable for X-ray tomography absorption contrast, as noted by Kuan et al. (2020). These tags, when fused with specific proteins, enable researchers to selectively label and study the dynamics of organelles, membrane structures, and the localization of proteins within cells in 3D space.

Computational processing in a High-Performance Computing (HPC) environment imposes several challenges, including storage of large datasets and models, memory capacity, and parallelization of algorithms (Zhang et al., 2023). Although deep learning techniques have been demonstrated as a cornerstone approach for image analysis, the use of such algorithms on large-scale datasets in HPC environments still requires advanced expertise in the design of parallel algorithms and programming in specialized language programming (e.g., C/C++, CUDA). To overcome this limitation, a new research area, called High-Performance Machine Learning, has recently emerged to provide methodologies and tools that explore data and model parallelism in a heterogeneous computing environment, i.e., composed of hundreds of CPU cores and GPUs, transparently to the users (Website, no date). Thus, the researchers can focus efforts on solving the problem by designing proper algorithms, without caring about model size and how to feed the neural networks with large datasets.

Another crucial limitation of deep learning techniques is their dependency on labeling data. Machine learning has streamlined the manual processing of imaging data, yet the scarcity of validated annotated datasets are bottlenecks. Collaboration within neuroscience is vital for creating integrated, standardized and multiscale validated datasets, akin to efforts by the Allen Institute. The demand for multidisciplinary experts in neurosciences and computational vision is rising to evaluate machine learning model predictions. Synthetic data generated by artificial intelligence serves as a data augmentation resource, mitigating the scarcity of labeled data in deep learning training. Vision transformers and morphological features for neuron classification are reshaping image analysis, enhancing algorithm performance, particularly with large datasets, and providing efficient methods to quantify mesoscale connectivity.

## 5 Conclusion

To attain a comprehensive understanding of brain function it is essential to seamlessly integrate cellular functions into the broader framework of brain organization. This integration involves incorporating fine details, ranging from the intricacies of dendritic spines to the branching patterns and interactions of individual neurons, into tridimensional models of neuronal network formation and adaptation to stimuli. A critical aspect of this integration is the preservation of the hierarchical organization of brain tissue, ensuring that cellular and sub-cellular data become an intrinsic part of the entire network. The mesoscale (cell-cell interactions) information links the micro/nanoscale (cellular and subcellular data) to the macro scale (whole brain functioning network). As such, integrative data can retrieve meaningful connections between cells, providing deeper insights into the complex neural network and reveal mechanisms underlying neurological and psychiatric disorders. Our review article aimed to highlight the state-of-the-art of the innovative field of neuroimaging in the context of the mesoscale, giving particular attention to its importance for a better comprehension of schizophrenia and epilepsy. This work presented the main techniques for image acquisition, data processing, and analysis optimized for mesoscale, emphasizing their distinctive aspects in analyzing specific structures, as well as acknowledging their limitations, especially concerning sample integrity. In this regard, we pinpoint multimodal imaging techniques like CLEM are emerging as the next frontier to capture large volumes in fine detail. Additionally, the latest (4th) generation of synchrotron accelerators offers approximately 1000x faster measurement capacities, enabling objective data generation through scanning, volume registration studies, and increased sample sizes (Winding et al., 2023).

Like every frontier of knowledge, neuroimaging is continuously expanding and experiencing rapid innovations. Its interdisciplinarity should not, therefore, be the primary limitation to its advancement. It is crucial that neuroscientists and computer scientists can comprehend the uses and potentials within the field of neuroimaging through a shared language.

## Author contributions

AC: Conceptualization, Writing – original draft, Writing – review and editing. AA: Conceptualization, Writing – original draft, Writing – review and editing. MA: Conceptualization, Writing – original draft, Writing – review and editing. BS: Conceptualization, Writing – original draft, Writing – review and editing. JR: Writing – original draft, Writing – review and editing. CC: Writing – review and editing. MF: Writing – review and editing. AP: Supervision, Writing – review and editing. LP-S: Writing – review and editing. SA: Conceptualization, Supervision, Writing – original draft, Writing – review and editing. MC: Conceptualization, Supervision, Writing – review and editing.

## Figures and Tables

**Table:** 

Adaptive Boosting (AdaBoost):	is a meta-learning algorithm that combines weak learners, slightly outperforming random guessing, into a weighted sum during the training process (Schapire and Singer, 1999).
3D neural organoids:	three-dimensional structures generated from stem cells that can mimic certain aspects of the developing human brain (Paşca et al., 2022).
Calcium-modulated photoactivatable ratiometric integrator (CaMPARI):	a photoconvertible calcium indicator to investigate network dynamics. CaMPARI shifts from green to red fluorescence when exposed to calcium influx and violet light, allowing for precise identification of activated neuronal populations (Fosque et al., 2015).
Confocal microscopy:	light microscopy technique that uses a laser to illuminate a single plane eliminating out-of-focus light to produce sharp, high-contrast images (Jonkman and Brown, 2015).
Convolutional Neural Network (CNN):	neural network architecture for images that uses a hierarchical stack of convolutional layers to extract features (Yamashita et al., 2018).
Covariance:	statistical tool used to understand the relationship between two variables. A positive covariance means both variables increase together, while a negative covariance suggests that they move in opposite directions (Koch et al., 1982).
DAPI (4′,6-diamidino-2-phenylindole):	fluorescent probe that binds to the minor groove (A-T rich) of double-stranded DNA (Kapuscinski, 1995).
Diffusion weighted image (DWI):	MRI technique that measures water diffusion in tissues. DWI images are used to diagnose and monitor a variety of neurological disorders (Baliyan et al., 2016).
Diffusion MRI tractography:	allow assessing the topological organization of brain networks (Jeurissen et al., 2019).
Diffusion Tensor Imaging (DTI):	MRI-based technique that measures the diffusion of water molecules within the brain. DTI and tractography are employed to visualize and map the pathways of nerve fiber bundles and the white matter tracts (Alexander et al., 2007).
Dijkstra algorithm:	greedy graph algorithm for finding the shortest path from a single source node to all other nodes in a weighted graph (Dijkstra, 1959).
Effective connectivity:	the causal interactions among neural elements within a neural system aiming to identify their intricate interrelation (Stephan and Friston, 2010).
Electron microscopy (EM):	technique that uses electron beams instead of visible light to achieve much higher resolution in imaging (Hayes and Pease, 1968).
Epileptogenic zone:	macroscopic/microscopic brain lesion responsible for the generation of seizures (Jehi, 2018).
Fast Marching Method (FMM):	numerical method for finding the global minimum of an energy function (Sethian, 1996).
Fields Of View (FOV):	in optical instruments is the angle at the camera’s vertex, created by the two edges that represent the maximum range within which the measured target’s object can traverse (Gao S. et al., 2022).
Filament tracing:	bioimaging technique used to reconstruct the three-dimensional (3D) morphology of neuronal filaments, such as axons and dendrites (Østerlund et al., 2022).
Fractional Anisotropy (FA):	metric in diffusion imaging that measures the directionality and integrity of water diffusion in tissues. Higher FA values indicate healthy white matter, while lower values suggest disruption or damage (Zolkefley et al., 2021).
Functional connectivity:	measurement of the synchronization of neural activity between different brain regions (Biswal et al., 1995).
GABA:	main inhibitory neurotransmitter in the brain, reduces neuronal excitability by binding to GABA receptors on neurons (Möhler, 2012).
Genetically Encoded Calcium Indicator (GECI):	a molecular probe designed to monitor intracellular calcium ion (Ca^2+) levels within living cells (Oh et al., 2019).
Generative Adversarial Networks (GAN):	type of deep learning model consisting of two neural networks, a generator and a discriminator, trained in a competitive manner to generate realistic data (Goodfellow et al., 2014).
Graph:	data structure composed of nodes or vertices connected by edges or links, representing their relationships (Zhou et al., 2020).
Hippocampus:	region of the brain located in the medial temporal lobe and involved in processes related to memories (Squire et al., 2004).
Ictal event:	seizure caused by abnormal electrical activity in the brain (Fisher et al., 2014).
K-Means clustering:	unsupervised learning technique that subdivides data into groups based on their characteristics. This algorithm operates by placing so-called centroids within the data’s vector space, and data points are classified by the centroid closest to them (Jin and Han, 2011).
L-measure:	software tool for quantifying the morphology of neurons (Scorcioni et al., 2008).
Light sheet microscopy or Selective Plane Illumination Microscopy (SPIM):	imaging technique that uses a thin sheet of laser light to illuminate a specimen from the side, minimizing photodamage and allowing for fast 3D imaging (Hillman et al., 2019).
Magnetic resonance imaging (MRI):	non-invasive, non-ionizing, quantitative, and multi-parametric imaging technique (Liu et al., 2021). It includes functional MRI (fMRI) and structural MRI (sMRI) (Shenton et al., 2012; Wu et al., 2021).
MCC-Net:	deep learning model for medical image segmentation that learns robust features at different scales of the image using contrastive learning (Wei et al., 2023).
Microtubules:	tube-like structures that provide structural support and facilitate intracellular transport (Desai and Mitchison, 1997).
Mossy fiber:	excitatory axons that project to and modulate hippocampal activity for spatial memory formation and consolidation (Henze et al., 2000).
MRI-T1:	type of MRI scan that produces images with high contrast between water and fat (Chen et al., 2018).
Neuropil:	defined as the space between neuronal and glial cell bodies that is composed of dendrites, axons, synapses, glial cell processes, and microvasculature (Spocter et al., 2012).
Point spread function (PSF) deconvolution:	method that attempts to correct the optical distortion. It serves as a mathematical representation of the blurring effect that occurs when a point source of light is recorded by an optical system (Corle and Kino, 1996).
Positron emission tomography (PET):	medical imaging technique that uses small amounts of radioactive tracers to visualize and measure physiological processes in the body (Phelps, 2000).
Principal Component Analysis (PCA):	statistical method for reducing the dimensionality of data while preserving as much of the variation in the data as possible (Jolliffe, 2002).
Pyramidal neurons:	Neurons exhibit a distinctive cellular structure, featuring apical and basal dendritic trees, as well as a pyramidal-shaped soma (Spruston, 2008).
RandomForest:	machine learning algorithm used for classification and regression, where the result is generated from the combination of multiple decision trees. Each decision tree is an independent model that divides the data into subsets based on a sequence of rules for data features (Belgiu and Drăguţ, 2016).
Regional homogeneity (ReHo):	concept and analysis technique to measure the similarity or coherence of the blood oxygen level-dependent (BOLD) signal within a specific brain region or voxel (Xu et al., 2019).
Region of interest (ROI):	significant portion of an image to be focused on or analyzed (Poldrack, 2007).
Residual Network (ResNet):	neural network architecture designed to address the vanishing gradient problem in very deep convolutional neural networks. It introduces skip connections, which allow gradients to flow more easily during training, improving the training of deeper networks in tasks like image recognition (He et al., 2015).
Resting state:	state in which a person is awake and alert but not actively engaged in a specific task or mental activity, also known as a baseline functional connectivity, when the brain continues to exhibit spontaneous neural activity (Biswal et al., 1995).
Serial section electron microscopy:	EM technique that sections and images a specimen to create 3D models of its internal structures, aiding in mapping neuronal connections within the brain (Gay and Anderson, 1954; Motta et al., 2019).
Sholl descriptors:	rule that links each neuron to a specific feature, associating neuron morphology with a function that produces numeric values in a metric space (Khalil et al., 2021).
Signal-to-Noise Ratio (SNR):	measure used to quantify the quality or strength of a signal in relation to the level of background noise or interference. A higher SNR indicates a more reliable and discernible signal compared to unwanted or random variations (noise) (Welvaert and Rosseel, 2013).
Single-photon emission computed tomography (SPECT):	nuclear medicine imaging technique that uses gamma-ray-emitting radiotracers to create 3D images of the distribution of radioactive compounds within the body (Devous, 1995).
Stimulated Emission Depletion (STED):	super-resolution microscopy technique that uses a combination of laser beams to overcome the diffraction limit, achieving extremely high spatial resolution in imaging (Vicidomini et al., 2018).
Structural connectivity:	Anatomical connections between different brain regions. It represents the physical pathways formed by bundles of nerve fibers (axons) that connect neurons in one part of the brain to neurons in another part (Hagmann et al., 2008).
Support Vector Machines (SVM):	supervised machine learning algorithm used for classification operates by identifying a hyperplane that separates different classes with a larger margin (Cristianini and Ricci, 2008).
Synchrotron x-ray microtomography:	non-destructive image technique that uses intense x-ray beams generated by a synchrotron particle accelerator to create high-resolution, 3D images of the internal structures of biological specimens (Betz et al., 2007).
Synthetic data:	artificially generated data used for various purposes, including training machine learning models (Rajotte et al., 2022).
Two-photon microscopy:	microscopy technique able to image a cm-thick biological specimen to create 3D images of its internal structures, aiding in mapping neuronal connections within the brain (Denk et al., 1990).
UNet:	Architecture employed for image segmentation. Uses convolutional layers for feature extraction and employs upsampling and transposed convolutional layers to create a segmentation mask (Ronneberger et al., 2015).
Voxel:	three-dimensional pixel, which is the smallest unit of a 3D space in a digital image or a 3D dataset (Foley, 1990).
Vision Transformers (ViT):	neural network architectures that utilize attention mechanisms to transform image patches into embedded representations, replacing traditional convolutional layers in computer vision tasks (Khan et al., 2022).
XGBoost (eXtreme Gradient Boosting):	an optimized version of the Gradient Boosting algorithm for classification and regression problems (Mason, no date).
Watershed:	image segmentation and instance labeling algorithm. It starts by dropping seeds to mark different regions, then these labeled seeds expand and delimitate the image into different areas, helping to identify individual instances in the image (Najman and Schmitt, 1994).
